# Network Analysis as a Tool to Understand Comorbidity Over Time

**DOI:** 10.1192/j.eurpsy.2026.10221

**Published:** 2026-07-31

**Authors:** A. C. Zegarra, D. E. Prieto-Molinari, B. Canessa-Lohmann, A. L. Delgado-Tenorio

**Affiliations:** 1 Faculty of Psychology; 2 Department of Psychopedagogical Guidance, Universidad de Lima, Lima, Peru

## Abstract

**Introduction:**

Network analysis provides a methodological alternative to traditional latent variable approaches by conceptualizing psychiatric disorders as systems of interacting symptoms. While cross-sectional network studies map comorbidity at a single point in time, repeated measures are needed to understand how these structures evolve. Longitudinal and repeated cross-sectional designs offer distinct strategies: the former tracks intra-individual symptom dynamics, while the latter captures population-level changes across independent samples. Methodological guidance on how to apply network analysis to comorbidity over time remains scarce, despite increasing demand to study the impact of contextual factors such as pandemics or socioeconomic stressors.

**Objectives:**

Demonstrate methodological strategies for applying network analysis to the study of comorbidities across time, and to illustrate their application with a repeated cross-sectional analysis of depressive symptoms and alcohol use disorder.

**Methods:**

We outline a stepwise framework for temporal network studies of comorbidity. Key stages include: (1) design selection (cross-sectional, repeated cross-sectional, longitudinal), (2) consistent measurement of symptom sets across time, (3) network estimation using regularized models, (4) evaluation of central and bridge symptoms with indices tailored to cross-disorder connectivity, (5) assessment of accuracy and stability via bootstrapping, and (6) network comparison across time points with formal tests of structural invariance. As an applied example, we employed a repeated cross-sectional study using Peruvian survey data on depressive symptoms and alcohol use disorder between 2018 and 2023, with more than 30,000 cases in each year.

**Results:**

he framework enabled detection of stable within-disorder symptom clusters and identification of bridge nodes linking depression and alcohol use. In Peru, worthlessness and guilt consistently emerged as central depressive connectors, while withdrawal acted as a key alcohol-related bridge. Cross-disorder associations intensified in 2020 during the COVID-19 pandemic, illustrating how external stressors can reshape comorbidity structures. Formal comparison confirmed overall invariance across years, highlighting both the sensitivity of the approach and the boundaries of repeated cross-sectional designs.

**Image 1:**
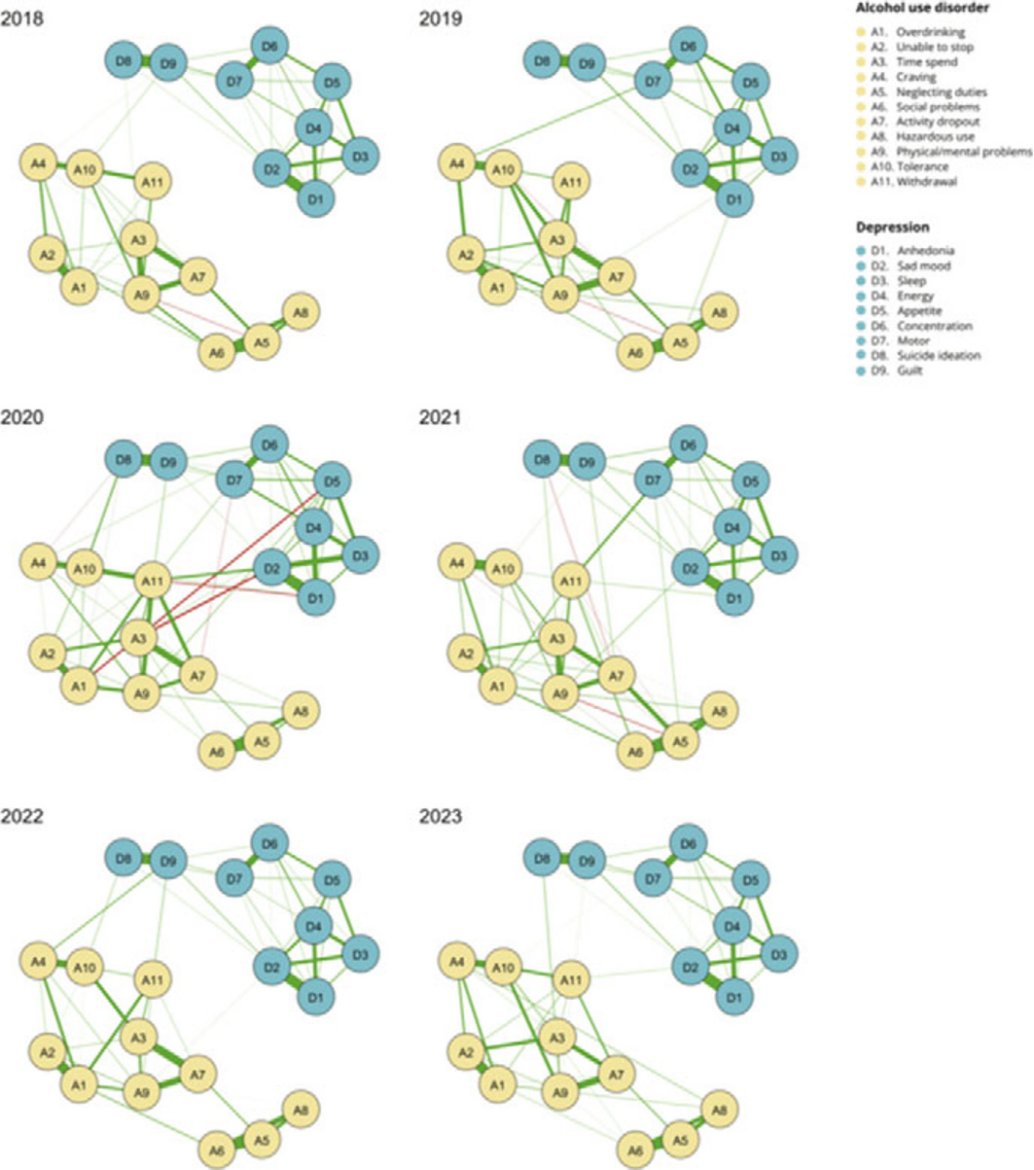
Image 1: Long description.

**Conclusions:**

The methodological guide demonstrates how temporal network analysis supports identification of core and bridge symptoms in comorbidity. Repeated cross-sectional designs capture population-level responses to contextual stressors, while maintaining feasibility at scale. This approach equips researchers with a practical roadmap to design, estimate, and interpret comorbidity networks across time, offering actionable insights for clinical and public health interventions.

**Disclosure of Interest:**

None Declared

## Long descriptions

### Image 1: Long description

Six network graphs arranged in a two-column by three-row grid represent years 2018, 2019, 2020, 2021, 2022, and 2023. Each graph contains two clusters of nodes.

Alcohol use disorder cluster (yellow nodes A 1 through A 11):

- A 1. Overdrinking

- A 2. Unable to stop

- A 3. Time spend

- A 4. Craving

- A 5. Neglecting duties

- A 6. Social problems

- A 7. Activity dropout

- A 8. Hazardous use

- A 9. Physical/mental problems

- A 10. Tolerance

- A 11. Withdrawal

Depression cluster (blue nodes D 1 through D 9):

- D 1. Anhedonia

- D 2. Sad mood

- D 3. Sleep

- D 4. Energy

- D 5. Appetite

- D 6. Concentration

- D 7. Motor

- D 8. Suicide ideation

- D 9. Guilt

In all years, nodes within each cluster are densely connected by green lines of varying thickness, indicating positive correlations. The strongest internal connections in the yellow cluster are between A 1, A 2, A 3, and A 9. In the blue cluster, D 1, D 2, D 3, and D 4 show high density.

Temporal changes:

- 2018 to 2019: The clusters remain largely separate with very thin, sparse bridge connections.

- 2020: A significant increase in cross-cluster connectivity occurs. Red lines, indicating negative correlations, appear between A 3 and D 1, D 2, and D 4. A 11 acts as a central bridge node between the two groups.

- 2021 to 2023: The density of cross-cluster connections decreases compared to 2020, returning to a state where the two symptom groups are more distinct, though A 11 remains a primary point of proximity between the clusters.

Navigate back to Image 1:.

